# Supplementary material for: Mapping Inter-individual Functional Connectivity Variability in TMS Targets for Major Depressive Disorder
Source: Front Psychiatry. 2022 Jun 23;13:902089. doi: 10.3389/fpsyt.2022.902089 (PMC9260048; doi:10.3389/fpsyt.2022.902089)
Supplement: Supplementary file 1 [file Data_Sheet_1.pdf]

Supplemental Information

Effect of varying E-field thresholds on the network engagement of TMS targets.

We use E-field thresholds in order to determine how much of the underlying cortical tissue is being activated by TMS stimulation. This is something that is not clearly understood, and moreover, the E-field threshold can vary across individuals (due to unique cortical geometry) and can also vary based on the experimental paradigm that is being used (excitatory vs. inhibitory TMS). Our default E-field threshold choice was 0.9 V/m, for reasons explained in the *Discussion*. However, in order to better understand the stability of our results across a range of potential threshold values, we further investigated the network engagement of TMS targets over a specific set of E-field thresholds ranging from 0.7 V/m to 0.9 V/m in increments of 0.05 V/m for a total of 5 different potential E-field thresholds. Our results showcase the effects of larger E-fields on the network engagement of TMS targets as expected, and are highlighted below.

E-field size and distribution

At the original threshold of 0.9 V/m, the E-field size ranged from 12 to 112.5 mm^2 (mean = 54.3 ± 18 mm^2) in the dlPFC and from 3.2 to 60.7 mm^2 (mean = 16.2 ± 8.5 mm^2) in the OFC. As noted in the *Methods*, E-field sizes are reported in terms of 'mm^2', where the average area of the face associated with vertex triplets is 0.05 mm^2. As we lowered the E-field threshold, the E-field sizes were seen to increase in both the dlPFC and OFC. At the lowest threshold value we looked at (0.7 V/m), the dlPFC E-field size ranged from 45.5 to 237.0 mm^2 (mean = 124.2 ± 33 mm^2). The OFC E-field size ranged from 18.6 to 130.1 mm^2 (mean = 44.5 ± 14.8 mm^2). Furthermore, in both the dlPFC and OFC, lowering the E-field threshold led to the spatial distribution of the respective E-fields reaching other areas of the cortex that were not the intended TMS target, and are unlikely to receive supra-threshold stimulation. For example, in the dlPFC, step-wise lowering of the E-field threshold from 0.9 V/m to 0.7 V/m saw the inclusion of parts of the motor and somatosensory cortex. On the other hand, in the OFC, lowering the E-field threshold led to the inclusion of parts of the dlPFC as part of the OFC E-field. These differences are showcased in Supplemental Figure 1.

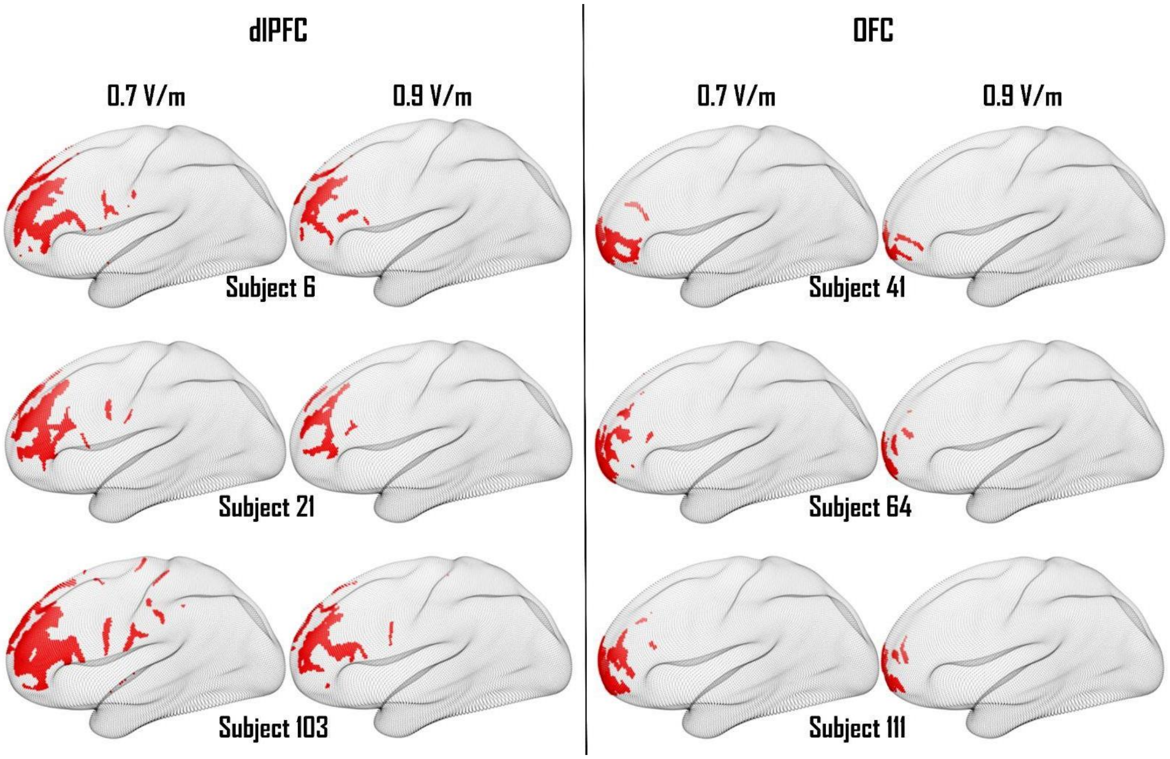

Supplemental Figure 1: The difference in the size and spatial extent of dlPFC and OFC E-fields are seen at the lowest (0.7 V/m) and highest (0.9 V/m) E-field thresholds.

Network engagement of TMS targets across a range of E-field thresholds

We observed that our results were highly stable across the various E-field thresholds, with one interesting exception.

The VAN, FPN, and DMN were seen as the major networks engaged from the dlPFC. The VAN engagement was seen to increase as the E-field threshold was increased. At the lowest E-field threshold (0.7 V/m), the VAN showed the highest engagement of all the networks in only 5 subjects (~4%). However, this number was seen to increase to 21 subjects (~17%) as the E-field threshold was increased to 0.9 V/m. We observed that the FPN was the most engaged network from the dlPFC in the majority of subjects, regardless of the E-field threshold value. At a threshold of 0.7 V/m, 92 of the 121 (~76%) subjects had the highest network engagement with the FPN. As the threshold was increased to 0.9 V/m, this number dropped to 81 (~67%). The DMN network engagement was relatively consistent across the different E-field thresholds. Maximum DMN engagement ranged from 16 subjects (~13%) at 0.7 V/m to 13 subjects (~10%) at 0.9 V/m (***Supplemental Figure 2A***).

In the OFC, we noticed an interesting pattern of network engagement between the FPN and DMN. As we increased the E-field threshold from 0.7 V/m to 0.9 V/m, there was a steady decrease in

FPN engagement and a steady increase in DMN engagement. At 0.7 V/m, the FPN was the maximally engaged network in 91 of 121 subjects (~75%), but at 0.9 V/m, this value dropped to 56 subjects (~46%). On the other hand, maximal DMN engagement was seen in 24 subjects (~20%) at 0.7 V/m but this number increased to 56 subjects (~46%) at 0.9 V/m matching the value for the FPN. Thus, the maximally engaged network (FPN vs. DMN) for OFC stimulation depends on the E-field threshold level (***Supplemental Figure 2B***).

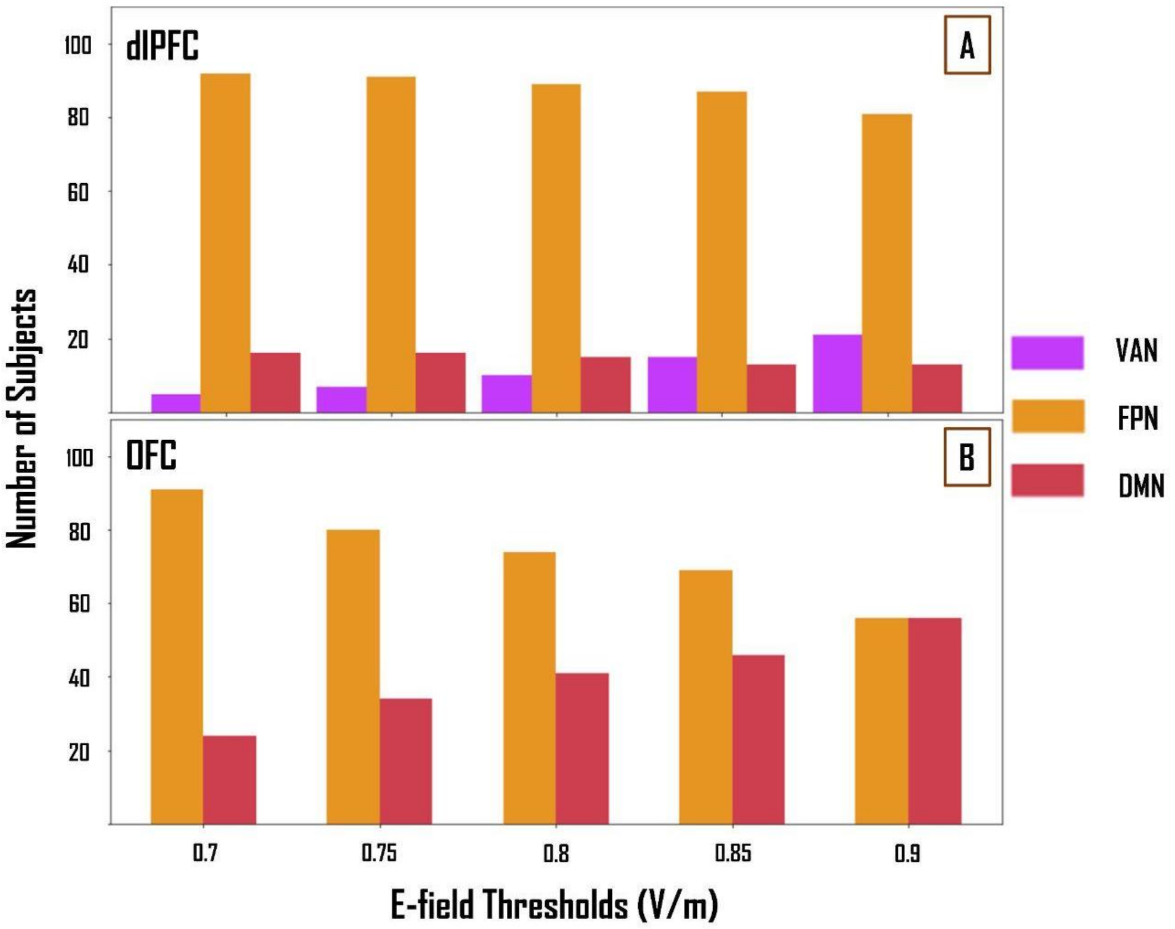

<u>Supplemental Figure 2:</u> **The network engagement of the VAN, FPN, and DMN are shown across multiple E-field thresholds across subjects. In the dlPFC, the FPN is the most engaged network across a majority of subjects regardless of the E-field threshold. In the OFC, the FPN engagement decreases, and the DMN engagement increase as the E-field threshold is increased, across subjects.**

Implication of findings

In addition to the main analysis of this paper, we looked at the effect of varying E-field thresholds on the network engagement of TMS targets. As expected, the overall E-field size and distribution were inversely proportional to the E-field threshold.

In the dlPFC, we observed that at lower thresholds, the E-fields included regions that were not the intended TMS target like the primary motor and somatosensory cortices. However, this didn't affect the overall network engagement drastically. The FPN was the most engaged functional network in a majority of subjects regardless of the E-field threshold. By increasing the E-field threshold (i.e., reducing the E-field size), we noticed that the VAN saw a slight increase as the most engaged network in some subjects. This could be due to the elimination of vertices on the more lateral/ventral surface of the PFC which are mainly associated with the FPN. The DMN was not the maximally engaged network across most subjects as the dlPFC E-field primary lies on the lateral/dorsal surface of the PFC while the DMN nodes are mainly more medial in location.

In the OFC, we observed an interesting relationship between the E-field threshold and corresponding network engagement of the FPN and DMN. As the E-field threshold was increased, we report that the FPN engagement was reduced while the DMN engagement was increased. The reason for this observation is evidenced directly from the E-fields themselves. At lower E-field thresholds (larger E-fields), the vertices of the E-field include regions of the dlPFC as part of the OFC E-field. As seen in our results above, dlPFC E-fields tend to maximally engage the FPN in a majority of the subjects. Therefore, this spill-over of E-field vertices from the OFC E-field leads to the inclusion of the dlPFC areas and thus a higher FPN engagement. Conversely, by increasing the E-field threshold (smaller E-fields), these additional dlPFC vertices are trimmed and this leads to a lower FPN engagement and a higher DMN engagement.

As noted in the main manuscript, we would again like to highlight the fact that our results show equal targeting of the FPN and DMN from the OFC at our original threshold (0.9 V/m). However, the DMN is engaged in a higher number of subjects from the OFC, than from the dlPFC, but the extent to which each network is engaged over the other comes down to the individual's FC.

Additional Figures

The figures below are enlarged versions of Figures 2, 3, and 4 - panel B in the main text. They have been included for additional clarity on what these figures were trying to convey.

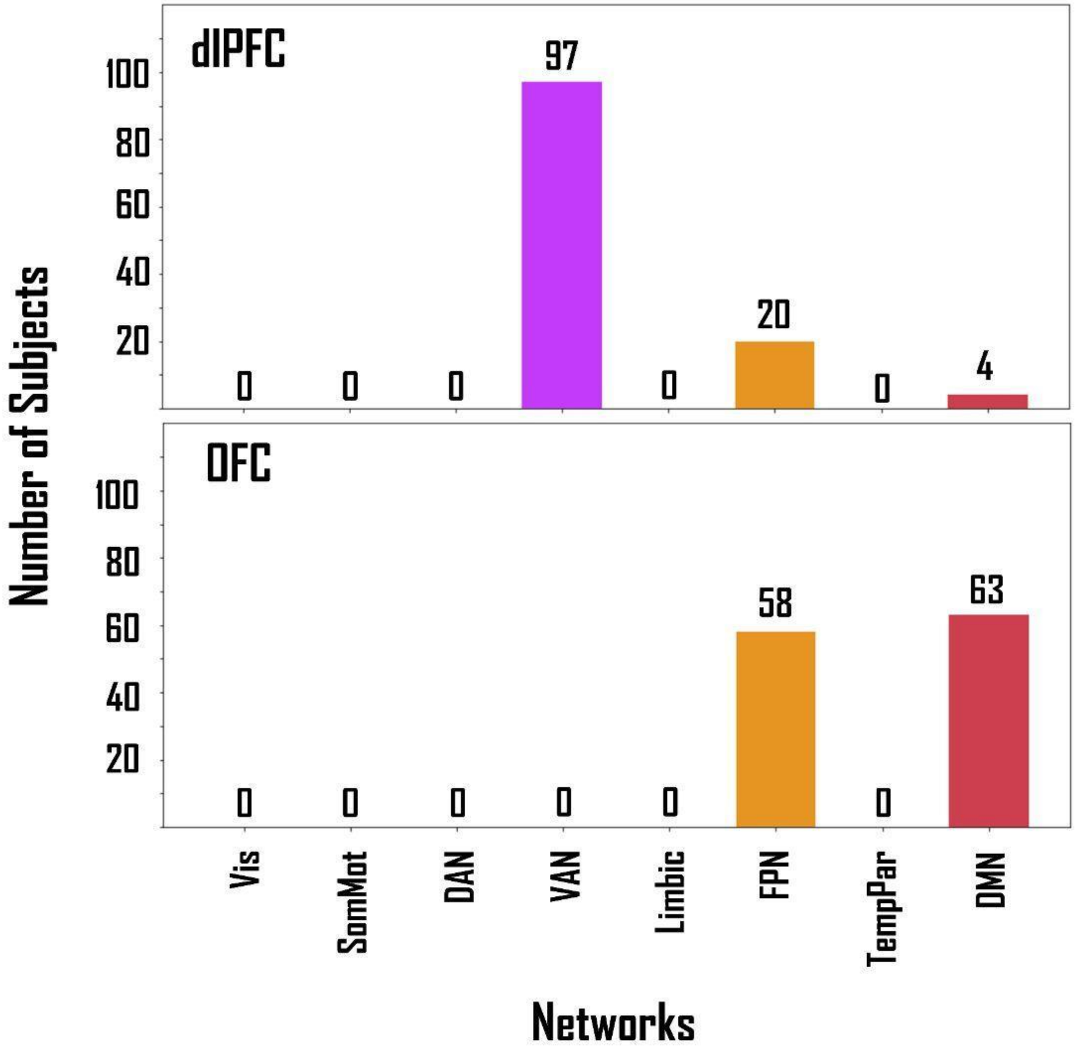

<u>Supplemental Figure 3:</u> Top: VAN, FPN, and DMN were the main networks engaged from the dlPFC (97, 20, and 4 subjects respectively). Bottom: FPN and DMN were the main networks engaged from the OFC (58 and 63 subjects respectively). Here we use subject-specific E-fields and the group average FC matrix. For more information refer to the *Results* section - Figure 2, Panel B.

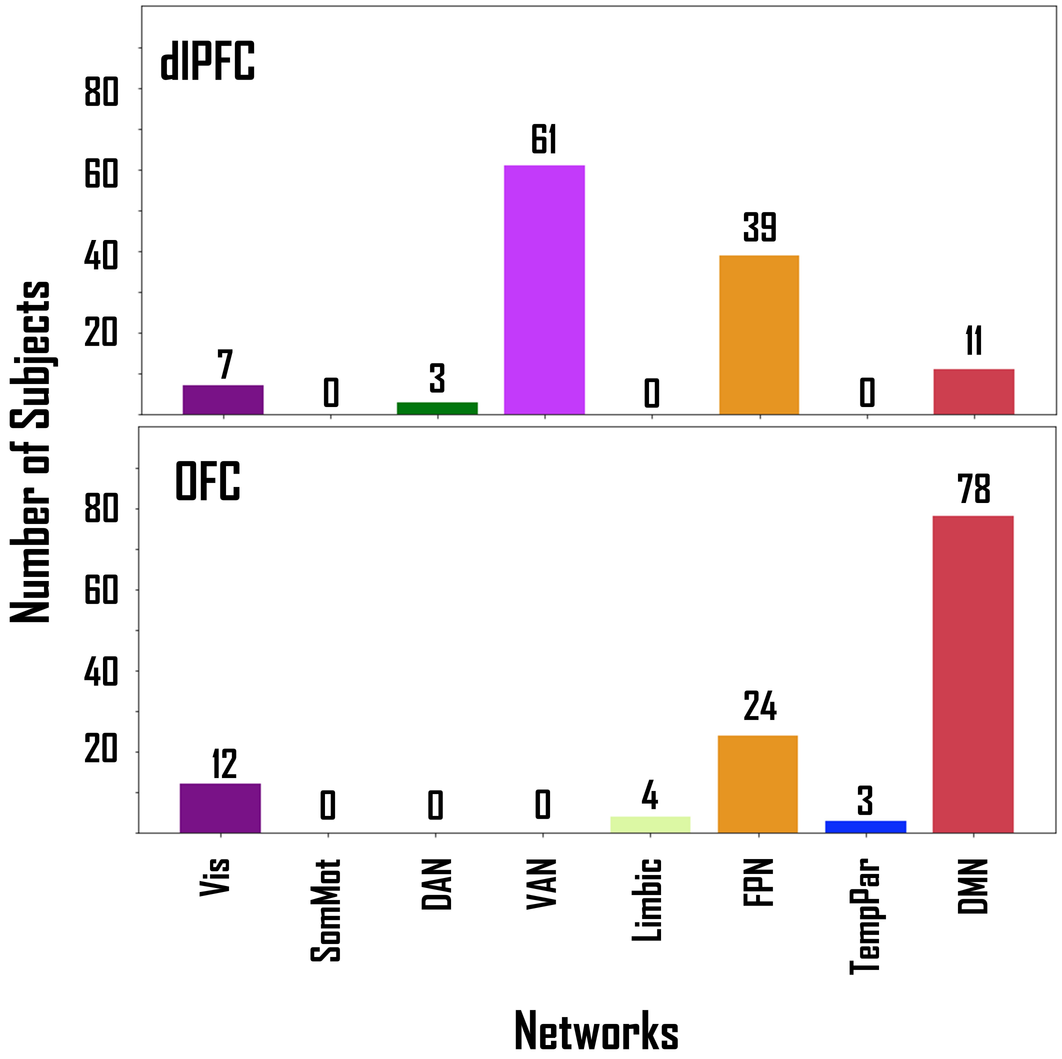

Supplemental Figure 4: **Top: VAN, FPN, and DMN were the main networks engaged from the dlPFC (61, 39, and 11 subjects respectively). Bottom: FPN and DMN were the main networks engaged from the OFC (24 and 78 subjects respectively). Here we use the Ernie E-field and subject-specific FC matrices. For more information refer to the *Results* section - Figure 3, Panel B.**

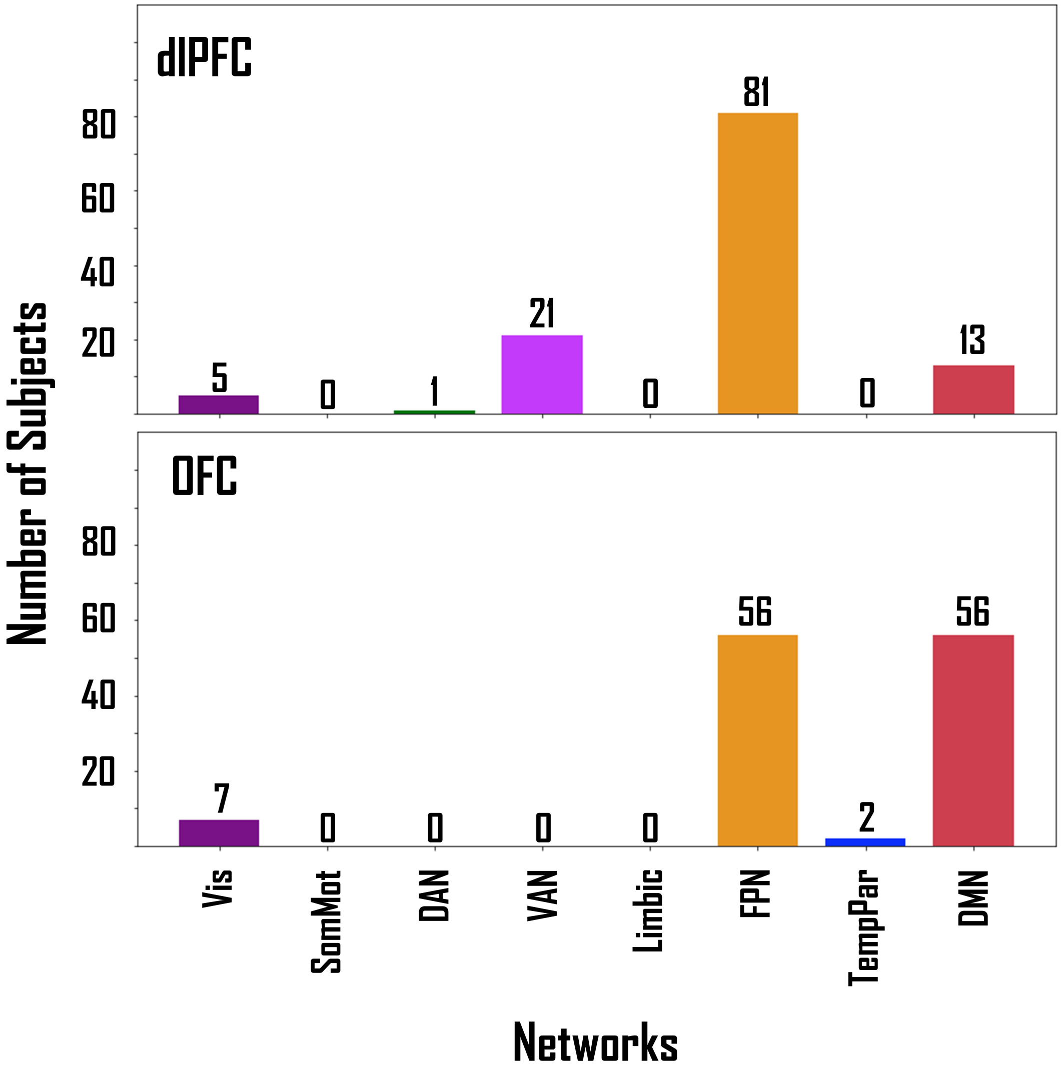

<u>Supplemental Figure 5:</u> Top: VAN, FPN, and DMN were the main networks engaged from the dlPFC (21, 81, and 13 subjects respectively). Bottom: FPN and DMN were the main networks engaged from the OFC (56 each). Here we use subject-specific E-fields and FC matrices. For more information refer to the *Results* section - Figure 4, Panel B.
